# Solvothermal Synthesis Combined with Design of Experiments—Optimization Approach for Magnetite Nanocrystal Clusters

**DOI:** 10.3390/nano11020360

**Published:** 2021-02-01

**Authors:** Joelle Medinger, Miroslava Nedyalkova, Marco Lattuada

**Affiliations:** Department of Chemistry, University of Fribourg, Chemin du Musée 9, 1700 Fribourg, Switzerland; joelle.medinger@unifr.ch

**Keywords:** magnetite nanocrystal clusters, solvothermal synthesis, design of experiment, response surface methodology, size control, optimization, superparamagnetism

## Abstract

Magnetite nanocrystal clusters are being investigated for their potential applications in catalysis, magnetic separation, and drug delivery. Controlling their size and size distribution is of paramount importance and often requires tedious trial-and-error experimentation to determine the optimal conditions necessary to synthesize clusters with the desired properties. In this work, magnetite nanocrystal clusters were prepared via a one-pot solvothermal reaction, starting from an available protocol. In order to optimize the experimental factors controlling their synthesis, response surface methodology (RSM) was used. The size of nanocrystal clusters can be varied by changing the amount of stabilizer (tribasic sodium citrate) and the solvent ratio (diethylene glycol/ethylene glycol). Tuning the experimental conditions during the optimization process is often limited to changing one factor at a time, while the experimental design allows for variation of the factors’ levels simultaneously. The efficiency of the design to achieve maximum refinement for the independent variables (stabilizer amount, diethylene glycol/ethylene glycol (DEG/EG) ratio) towards the best conditions for spherical magnetite nanocrystal clusters with desirable size (measured by scanning electron microscopy and dynamic light scattering) and narrow size distribution as responses were proven and tested. The optimization procedure based on the RSM was then used in reverse mode to determine the factors from the knowledge of the response to predict the optimal synthesis conditions required to obtain a good size and size distribution. The RSM model was validated using a plethora of statistical methods. The design can facilitate the optimization procedure by overcoming the trial-and-error process with a systematic model-guided approach.

## 1. Introduction

Recent decades have seen an increasing interest in magnetic nanoparticles because of their unique properties, which find potential applications in biomedicine, separation, and catalysis [1,2]. The most studied magnetic nanoparticles are those made of magnetite (Fe_3_O_4_). They show excellent magnetic responsiveness [3], have low toxicity [4] and high chemical stability, and can be made biocompatible when a suitable surface functionalization is selected [5,6]. In addition, a wide variety of synthetic methods to prepare magnetite nanoparticles in a broad range of sizes and shapes have been developed, some of which can be easily scaled up [1,2,3,4,5]. When the size of magnetite nanoparticles is smaller than ~25 nm, they display superparamagnetism [7]. Superparamagnetism can be very advantageous because it enables one to turn on and off their dipolar interactions, thus controlling their ability to form chains and to respond to magnetic field gradients [8,9]. In that size range, magnetite nanoparticles are in a single magnetic domain while still possessing high magnetization, which makes them respond very rapidly to an external applied magnetic field. When the field is removed, thermal motion randomizes the alignment of the magnetic moment and the particles show no magnetic behavior anymore. These nanoparticles are known to be ideal candidates for medicinal applications, for example, as MRI negative contrast agents [10,11], in cancer therapy [12,13], hyperthermia therapy [14,15,16], and diagnostics [17,18], or for targeted drug delivery [19,20]. The small size of these superparamagnetic particles can, nonetheless, also be a disadvantage, depending on the desired application. For example, manipulation of small particles can be difficult, as high external magnetic fields are necessary to induce a strong response because their magnetic moment is proportional to their volume. Further increasing the size of the nanocrystals will, however, lead to a change in magnetic behavior, from superparamagnetic to ferrimagnetic. In order to maintain their superparamagnetic behavior while increasing their size, one effective possibility is to enclose several small magnetite nanocrystals inside bigger particles, for example, in a polymer or inorganic particle, leading to superparamagnetic beads with sizes ranging from 50 nm to a few microns [21]. A similar strategy to increase their magnetic response without losing their superparamagnetic behavior is by forming clusters of pure magnetite nanocrystals in sizes ranging from 50 nm to 1 micron or even larger. These superparamagnetic nanocrystal clusters find applications in catalysis [22], responsive optical materials [23], and magnetic fluids [24], photonic crystals [25], and wastewater treatment [26,27]. To satisfy the requirements imposed by applications, the size of the primary nanocrystals, the size of the final clusters, and their shape and density must be controlled. It is especially important for nanocrystal clusters used in biomedical applications to be dispersible in water, to keep a spherical shape, and to have a narrow size distribution and a high colloidal stability.

Many different synthetic routes to synthesize magnetite nanocrystal clusters have been published, using different stabilizing agents, all leading to different size ranges depending on the composition of the system [25,28,29,30]. Choosing among all the available possibilities to reach the desired size, shape, biocompatibility, or polydispersity can be difficult. Even after having made a choice, the researcher can be confronted with a trial-and-error approach to reach the desired target. In this paper, an approach is presented to avoid this tiring and frustrating work. A model has been developed, based on experimental design, which allows scientists to choose the target size measured via dynamic light scattering (DLS) or scanning electron microscopy (SEM), the polydispersity index (PDI), also estimated from DLS, or the standard deviation of their size distribution measured from SEM images and returns an optimized recipe to synthesize the desired spherical magnetic clusters. Our work has been based on a well-known hydrothermal synthesis giving spherical clusters with well-controlled surface functionalization and size distribution. For our study, the synthesis method first published by Wang et al. [28] was chosen. The method uses anhydrous iron (III) chloride as an iron source, which is dissolved with trisodium citrate in a solvent mixture composed of diethylene (DEG) and ethylene glycol (EG), with the addition of anhydrous sodium acetate as an alkaline source. Different parameters of the synthesis can be changed to affect the final size of the clusters. First, the amount of trisodium citrate changes the clusters’ secondary size because it serves as the stabilizing agent for the nanocrystal clusters, while the ratio of DEG to EG also affects the overall size, since DEG and EG both play a role not only as solvents but also as reducing agents for the iron chloride. Similar protocols with urea or ammonia as precipitating agents instead of sodium acetate [31,32] or polyethylene glycol (PEG) [33] and polyacrylic acid (PAA) [25] as stabilizers instead of citric acid have been published. Citric acid has the advantage of coordinating strongly with the iron oxide surface with its three deprotonated acid groups, yielding highly water-dispersible particles. 

Response surface methodology (RSM) was used to test the effect of experimental factors, i.e., citrate concentration and solvent ratio, on the size and polydispersity index of nanocrystal clusters. The procedure makes it possible to assess the effect of each input factor and the interactions between them on the chosen response(s) to construct a regression model and to illustrate the input factors’ impact by means of a response surface (2D, 3D, or contour line). The RSM applicability for cluster synthesis can be very valuable, as it allows for a quick and effective selection of the optimal conditions and decreases trial-and-error runs [34,35,36]. 

**Figure 1:**
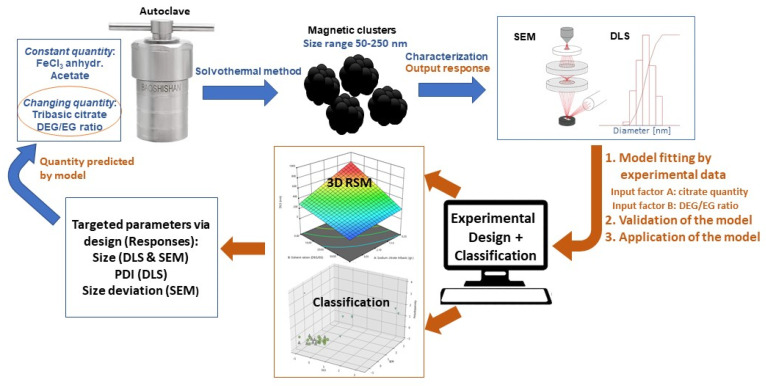
Schematic representation of the experimental and computational work to construct the model.

The present study uses an experimental–statistical approach for the optimized synthesis of magnetic iron oxide nanocrystal clusters. The combination of Design of Experiment (DOE) with supervised and unsupervised approaches is shown to be a proper path to reveal the factors affecting the responses, meaning the size and polydispersity, of the magnetic nanoparticles. Moreover, it highlights the limits in the variation of the factors for the targeted nanocrystal cluster size and the respective applied synthetic protocol. A graphical illustration of all the performed steps for this study is presented in Figure 1.

## 2. Materials and Methods 

Iron (III) chloride anhydrous >95%, sodium citrate tribasic dihydrate, and sodium acetate anhydrous were purchased from Sigma-Aldrich (Merck, Switzerland). Ethylene glycol > 99% and diethylene glycol >99% were also purchased from Sigma-Aldrich.

All reactions were carried out in Baoshishan hydrothermal synthesis autoclaves consisting of a high-pressure stainless steel container and a Teflon lining, with a total internal volume of 100 mL. The particles were characterized by means of DLS and zeta potential measurements (BECKMAN COULTERDelsa Max Pro, Brea, California USA), scanning electron microscopy (SEM) (TESCAN Mira 3 LM field emission, Dortmund Germany), and transmission electron microscopy (TEM) (FEI Tecnai Spirit field emission Hillsboro, Oregon, USA) to analyze their size (both in dry state and in solution) and their charge. MicroMag^TM^ of PCM (Princeton Measurement Corporation) vibrating sample magnetometry (VSM) was used to measure magnetization curves. 

We synthesized nanocrystal clusters with sizes ranging from 50 to 300 nm, stabilized with tribasic citrate to build up the model. The citrate concentration and solvent ratio were changed as shown in Table 1. In all syntheses, the DEG/EG ratio was changed, as reported in Table 1, while always keeping a total solvent volume of 80 mL. In a standard recipe, 1.62 g anhydrous iron (III) chloride and 1 g trisodium citrate were added to a 100-mL 3-necked round-bottom flask. Then, 80 mL of solvent with a DEG/EG ratio equal to 1 (meaning 40 mL/40 mL of DEG/EG) was added. Besides using anhydrous reagents, no specific precautions were taken to work under anhydrous conditions. The mixture was magnetically stirred at 500 rpm for 30 min at 120 °C in an oil bath. Subsequently, the heating was stopped and 4.1 g of sodium acetate were added to the mixture, which was vigorously stirred for another hour. Finally, the brown-yellowish mixture was poured into the Teflon-lined stainless steel autoclave. The autoclave was placed in an oven, where the reaction was carried out for 10 h at 200 °C. The obtained black precipitate, consisting of magnetite nanocrystal clusters with an average size of 150 nm, was washed via magnetic decantation. A first washing step was performed with distilled water, while a second washing step was performed with distilled water, the pH of which was increased to 10 (by means of NaOH) to promote deprotonation of the carboxyl groups on the surface of the clusters. In this manner, their electrostatic colloidal stability was enhanced and their aggregation was usually avoided. A small amount of tribasic citrate was added to the washing solvent to compensate for detaching citrate on the surface of the particles during washing. The washed nanocrystal clusters were eventually dispersed in 40 mL aqueous solution at pH 10.

If some aggregation in solution was detected by DLS after the sample was kept for longer time on the bench, 0.1–0.2 mg of tribasic citrate was added after the washing steps to the sample, followed by two minutes of sonication with a horn sonicator (Dr. Hielscher ultrasonic processor UP 400 s). This procedure was very effective at breaking up the aggregates and retrieving a well-dispersed sample. The clusters had a high density and were prone to sedimentation and aggregation. Especially those samples with a cluster size above 200 nm showed a higher risk of aggregation, already at 1 week after preparation. In these cases, sonication with a sonicating bath was sufficient to effectively redisperse them.

## 3. Results and Discussion

Monodisperse superparamagnetic magnetite nanocrystal clusters were produced via a solvothermal method. In the protocol used to synthesize the nanocrystal clusters, iron (III) chloride is reduced at 200 °C by diethylene and ethylene glycol, playing the roles of both reducing agents and solvents. Sodium acetate serves as an alkaline source and as a precipitating agent, while sodium citrate is used as stabilizer. The formation of the nanocrystal clusters follows a well-understood two-stage growth, starting from nucleation of magnetite nanocrystals and followed by their aggregation to form clusters [34]. First, primary-sized nanocrystals are formed, the so-called nuclei, which, after reaching a critical concentration, can overcome the energy barrier to agglomerate into nanocrystal clusters. We speak of primary size when referring to the nanocrystal size, while the secondary size describes the size of the secondary structure, meaning the nanocrystal clusters. The use of sodium citrate as a stabilizer leads to stable nanocrystal clusters in aqueous solutions, especially when the pH is high enough to have complete deprotonation of the three acid groups. Under such conditions, the nanocrystal clusters’ surface becomes hydrophilic.

In their systematic study, Wang et al. [28] showed that an increase in citrate concentration leads to smaller nanocrystal clusters, until a critical threshold is reached. In contrast, no addition of citrate led to non-uniform clusters, showing the influence of citrate on the morphology of nanocrystal clusters. They also showed that the solvent DEG/EG ratio also has an impact on the nanocrystal clusters’ size and size distribution. An increase in this ratio leads to smaller nanocrystal clusters, because DEG is more viscous than EG and forms a stronger coordination complex with iron. An increase in the DEG/EG ratio helps to reduce the surface energy of the primary crystals; in this case, more nuclei are obtained. The use of pure DEG leads to a high amount of very small particles, which will not properly agglomerate and have a non-uniform shape. This shows the importance of choosing the optimal solvent ratio for controlled secondary size and shape of the nanocrystal clusters. 

Because of the complex interplay between the concentration of citrates and the solvent composition, we decided to use experimental design in order to optimize the synthesis of the clusters. The idea was to use the synergy between the experimental data and a statistical-aided approach, based on response surface methods (RSMs), for the design of experiments (DOE) [37,38,39]. In this manner, we set out to find the optimal experimental environment for the synthesis of iron oxide nanocrystal clusters of a specific size and a low PDI. Next to the concentration of the stabilizer and the ratio of the solvent, the reaction temperature and the reaction time are prone to induce a change in the size of nanocrystal clusters. Because the temperature limit of the Teflon-lined autoclave is 220 °C, under which conditions Teflon already deforms, the temperature can only be decreased. At 180 °C, no clusters were produced. The same was observed when the reaction time was reduced first to 7 h and then to 8 h. Some clusters were obtained at a temperature of 200 °C, but the reaction was not complete. A reduced amount of clusters was obtained compared to the amount obtained after 10 h. The time was not increased above 10 h because the reaction time should be as low as possible for a useful and reasonable protocol.

Ultimately, the aim of the model is to target a specific nanocrystal cluster size and a specific polydispersity index, and the model will provide an optimal synthesis recipe as an output. Two input factors have been used: A, the concentration of citrate, and B, the DEG/EG ratio. The responses are the secondary size of the nanocrystal clusters, determined from DLS and SEM, as well as the polydispersity index (PDI) from DLS and the standard deviation of the size obtained from SEM image analysis. The last two quantities are used as a measure of how narrow the obtained size distribution is. To summarize: for a given target size, the model will give a certain solvent ratio and tribasic citrate amount, which should lead to the desired nanocrystal cluster size with a PDI as low as possible.

The Design Expert software^®^ (version 12, StatEase Minneapolis, Minnesota (MN), USA) was used for optimization and control of the nanocrystal cluster’s size by computationally aided nonmanufacturing design [40]. The proposed statistical approach is the most suitable to predict and tune the behavior of a complex system, such as the one investigated in this work, taking into account the dynamic complexity of the reaction factors (and some external factors) and their impact on the final responses. The model can be trained in order to respond to a multifactor design of experiments methodology. The plan of the experimental design is an advanced form of DOE that has been successfully tested with response surface methods as an optimization protocol for the factors (experimental parameters) involved in the one-pot synthesis of iron oxide nanocrystal clusters. A statistical analysis based on the application of the multivariate optimization approach was used on a set of various linear and polynomial models fitting the dependent variables. Based on an empirical quadratic polynomial (for our experimental set), the values of the dependent variable were predicted.

In order to define the model as significant or even very significant, an assessment and validation were conducted by means of analysis of variance (ANOVA). Furthermore, to evaluate the model’s applicability to the experimental data, the Fisher F-value (obtained from the F-test), the squared correlation coefficient (R^2^), the adjusted R^2^, and the R^2^ of predictions were calculated and integrated in the validation procedure to find the best regression model. Based on the diagnostic capabilities of the statistical criteria for the best model selection, two non-linear regression models were selected for the design space. On one side, a quadratic model for the first three responses (DLS size, SEM size, and standard deviation of SEM size measurements) was used, while a two-factor interaction model (2FI) for the fourth response (PDI) was adopted. Note that the four responses are dependent variables. Independent variables or factors are input variables from the experimental conditions and the dependent variables are defined as responses (measured effects). 

**Figure 2:**
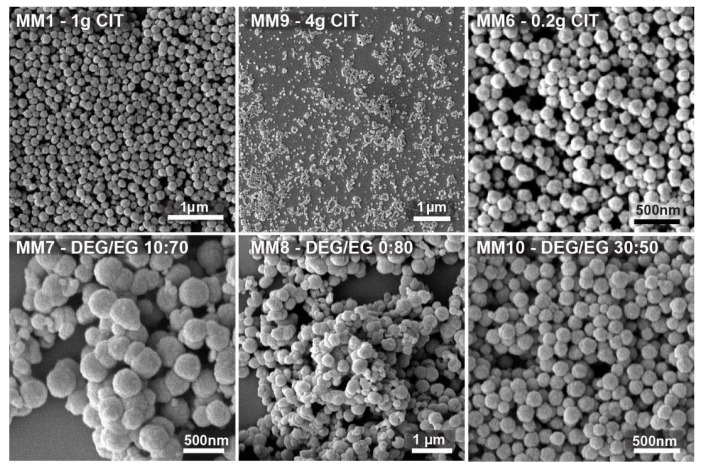
SEM images of the nanocrystal clusters prepared from the first set of experiments to check the system’s response to a change in quantities of tribasic citrate and diethylene glycol/ethylene glycol (DEG/EG) ratio, used to build up the model. The top three images show nanocrystal clusters prepared with a fixed ratio DEG/EG = 1 but with different citrate amounts. The bottom three images show the nanocrystal clusters prepared at a constant citrate amount equal to 1 g, but with different solvent ratios.

To begin, we performed a set of 18 experiments, out of which only 13 gave satisfying results, meaning they generated clusters with acceptable PDI and a spherical shape. Those 13 experiments were used to generate the model. In this test series, we either modified the citrate amount by keeping the solvent ratio constant and equal to 1, or we changed the solvent ratio by keeping the citrate amount constant at 1 g. In this way, we obtained nanocrystal clusters with sizes between 50 and 300 nm but with various degrees of polydispersity. We also tried to find the limits of the synthesis process, meaning the smallest and biggest nanocrystal clusters achievable with an acceptable polydispersity. Moreover, we tested which citrate amount or solvent ratio led to stable nanocrystal cluster dispersions. All of the nanocrystal clusters were characterized by both DLS and SEM. ImageJ software was used to extract the l cluster size from SEM pictures. Stabilizing the nanocrystal clusters with citrate led to a negative charge at the nanocrystal clusters’ surface. Measured zeta potential values were ranging from −10 to −35 mV, confirming the negative surface charge. In Figure S1, SEM images of three examples of clusters obtained from the experiments are shown, which were excluded from the dataset used to fit the model because either the polydispersity was very high or the nanocrystal clusters had poor sphericity, as can be observed, for example, from the SEM image of the sample MM31. In Figure 2, instead, a few SEM images are shown of the nanocrystal clusters prepared from the experiments used to fit the model. The data clearly show that the admissible interval of citrate quantity ranges from 0.2 to 4 g.

**Figure 3:**
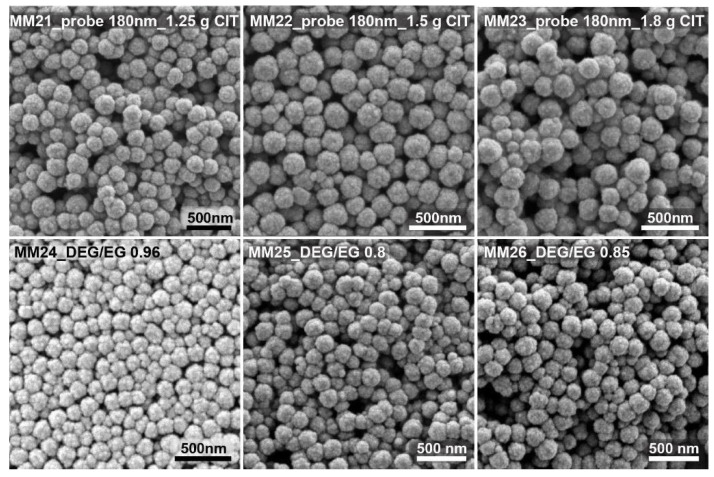
In the top three SEM images, nanocrystal clusters were prepared with a citrate amount varied between 1.25 and 1.8 g, with a constant DEG/EG ratio equal to 1. In the bottom three SEM images, nanocrystal clusters were prepared by changing the solvent ratio between 0.75 and 0.85, with a constant citrate amount of 3 g. The nanocrystal cluster size was found to be 180 nm plus/minus 20 nm.

**Figure 4:**
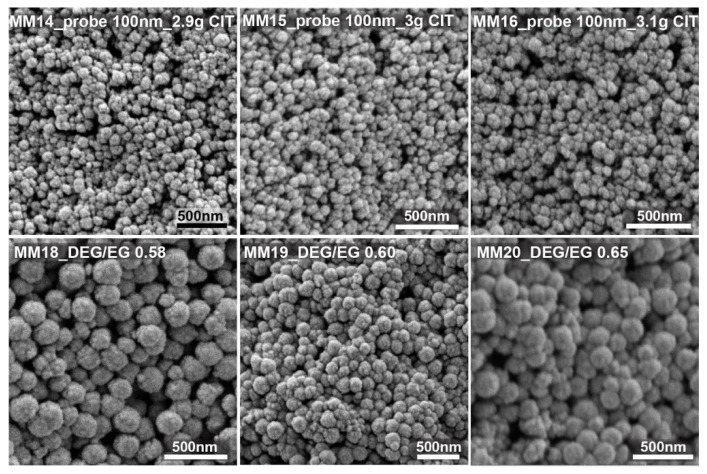
The top three SEM images show nanocrystal clusters prepared by varying the citrate amount between 2.9 and 3.1 g, with a constant DEG/EG ratio equal to 1. The bottom three images show SEM images of nanocrystal clusters obtained from different DEG/EG ratios but a constant citrate amount of 3 g.

MM1, with 1 g citrate, led to monodisperse 150 nm nanocrystal clusters. MM9 shows nanocrystal clusters of 50 nm. They were the smallest nanocrystal clusters that were synthesized with a citrate amount of 4 g and with an already quite high polydispersity index of 0.24. MM6 clusters, prepared with 0.2 g citrate, were the biggest clusters produced, still having an acceptable polydispersity of 0.2 and a size of 300 nm (DLS). The addition of lower or higher citrate amounts led to either colloidally unstable cluster dispersions or nanocrystal clusters with unacceptable low sphericity and/or unacceptable high polydispersity, respectively. The DEG/EG ratio was changed between 0 and 0.85. It was observed that the acceptable limits in terms of the polydispersity and sphericity of the obtained nanocrystal clusters are between 0.45 and 0.85. In MM7 and MM8, low DEG/EG ratios were used. It can be observed that the polydispersity is very high, and the particles clearly lose their spherical shape. These two experiments prove that the amount of diethylene glycol is important to produce monodisperse, spherically shaped clusters. MM10, prepared with a higher DEG/EG ratio of 0.6 shows monodisperse nanocrystal clusters with spherical shape and with a size of 200 nm. Overall, we produced nanocrystal clusters with a size range between 50 and 300 nm (DLS), and with a polydispersity index (PDI) between 0.01 and 0.55. After using the first batch of data to fit the model, a first iteration was performed to optimize the input factors and, consequently, the responses. This was realized by targeting nanocrystal clusters of about 180 and 100 nm in size (see Figure 3 and Figure 4). Seventeen recipes targeting the same nanocrystal cluster size but differing in terms of citrate quantity and solvent ratios were tested. Those runs’ measured PDI values figured within the PDI acceptable range. These 17 recipes were used together with the original 13 cases to optimize the design, resulting in 30 overall cases, which were the training set for the actual model. It is important to note that in the case of the optimization process, some experiments were repetitions of the initial 13 experiments, especially when targeting for a specific size. Those repetitions were conserved in the list of experiments given by the model. The repetitions had, on one side, the purpose of checking for the reproducibility of the experimental procedure used, and on the other hand, they confirmed that the model is working well, as the same recipe already used to prepare clusters with a certain size is given as an output. The collected 30 runs were designed by RSM. The full table with the experimental runs is presented in Table 1, with the corresponding responses for the size of nanocrystal clusters measured by dynamic light scattering as well as by SEM measurements. The full magnetization curve of some of the prepared clusters was measured by means of VSM, confirming the superparamagnetic behavior of the synthesized clusters. In Figure S2, three magnetization curves for three of the prepared samples are shown. The curves have been chosen as representative of small clusters, medium-sized clusters, and large clusters. All curves show no significant hysteresis, thus confirming the superparamagnetism of the clusters.

Figure 3 shows some of the SEM images of the test run, targeting nanocrystal clusters with sizes of around 180 nm. By changing the citrate amount from 1.25 to 1.8 g, clusters with sizes from 200 to 180 nm were obtained. Small changes in the citrate quantity could be used to fine tune the cluster size within a range of about 20 nm. Changing the solvent ratio from 0.75 over 0.8 to 0.85 yielded nanocrystal clusters with sizes ranging from 150 to 200 nm.

Figure 4 shows some of the SEM images of the nanocrystal clusters obtained from the test run, targeting clusters around 100 nm. For this case, the citrate amount was changed between 2.8 and 3.2 g, with a constant solvent ratio of 1. The SEM images confirm that the size changes are very small and that nanocrystal cluster size can be tuned from 98 to 120 nm. Changing the solvent ratio between 0.6 and 1.65 with 3 g of stabilizer resulted in magnetic nanocrystal clusters with sizes of 150 to 190 nm. 

It appears that a non-linear model is required to capture the observed trends. A quadratic model in the frame of RSM was used for the DLS, SEM, and standard deviation responses. For the fourth response, i.e., the polydispersity index, a two-factor interaction (2FI) regression model was adopted. The DLS, SEM, and standard deviation key responses were coded as *Y_i_* for the quadratic model based on the experimentally conducted data. *A* represents the tribasic sodium citrate amount in grams, while *B* indicates the solvent ratio (DEG/EG) for a total volume of solvent, being 80 mL. The quadratic model for this critical-to-optimization attribute is indicated below (Equations (2)–(4)):

(1) $$Y_{i}=\beta_{0}-\beta_{1}\left(A\right)-\beta_{2}\left(B\right)+\beta_{12}\left(A\right)\left(B\right)-\beta_{11}(A^{2})-\beta_{22}(B^{2})$$

(2) $$Y_{1}\left(DLS\right)=354.02-157.98\;A-203.76\;B+97.26\;AB-2.00\;A^{2}-2.28\;B^{2}$$

(3) $$Y_{2}\left(SEM\right)=299.57-104.09\;A-150.31\;B+51.89\;AB-44.68\;A^{2}-1.94\;B^{2\;}$$

(4) $$Y_{3}\left(\;SEM\;standard\;deviation\right)=30.74-24.26\;A-7.3\;B+20.63\;AB-10.55\;A^{2}-7.94\;B^{2}$$

The intercept in all the equations is labelled as *β*_0_. The interpretation of the intercept in the frame of this approach is an indication of the scaling behavior of the response (increasing/decreasing) divided by the factor level (two coded factors). The *β*_1_ coefficient is one-half of the value of the effect. The coefficient assessment represents the expected variance (change) in DLS, SEM, and standard deviation per unit variation in factor value when all residual factors are constant. Since the input factors are coded (normalized) as +1 and −1 (the range of values is from −1 to +1), the calculated coefficients represent the averages of each factor according to the used design.

The data for the variance inflation factor (VIF) can be found in the (supplementary information Tables S1–S4) showing the table of coefficients in terms of coded factors, together with an assessment metric for the variance of the model. If the factor is orthogonal to all the other factors in the model, the VIF is 1.0. A VIF greater than 1 indicates multicollinearity, and the higher the VIF, the more correlated the factors are within the set.

For the last response of the system, namely PDI, a 2FI regression model fits realistically, thereby adequately expressing the experimental range studied (Equation (5)).

(5) $$Y_{4}\left(PDI\right)=0.1599-0.3892A+0.0016B\;+0.4662AB$$

In Table S4 containing the coefficients in terms of coded factors, together with an assessment metric for the variance of the model, the VIF factor for the coded coefficients is estimated and represents the expected change in response per unit change in factor value when all remaining factors are held constant. As can be observed in Equations (2)–(5), the interaction terms AB are positive, implying a synergistic effect of the factors on the size of the nanocrystal clusters. However, it should be emphasized that the proposed models have a dual-factor parameter nature, so the effect of the responses and their interactions should be considered simultaneously. The extended regression diagnostic approach was proposed as an additional metric for the evaluation of the models’ accuracy. This has been conducted by comparing the normal probability of residuals with the internally studentized (standard deviations that separate the actual and predicted response values) residuals of the predicted response. With the externally studentized residual plots (Figures S3–S6), a model was tested for outliers by means of an outlier-t-test within the runs, assuming the chosen model holds. Within the explored systems, four levels indicated the outliers (runs MM6, MM7, MM8, and MM10). The model adequacy is also demonstrated in Figures S7–S10. The labeling of the plots as DFBETAS is for “difference in betas”. The “beta” is the symbol used for the coefficients in the model. The meaning of this diagnostic is to determine the influence of each run on a given coefficient. The design procedure was applied to the system by considering and checking for variance homogeneity, coefficients’ significance, and adequacy of the models. If the input factors are not correctly chosen and their interval of variation is not precisely set up, a pre-selection procedure is recommended. Thus, the pre-selection procedure becomes part of the design strategy. To conclude, the pillars for optimization include pre-selection of input factors, appropriate design scheme, construction of a regression model, statistical assessment of the models, and validation of the optimized synthetic process.

The results obtained from the application of the RSM were evaluated by ANOVA for the four responses and are shown in Table 2. From these four responses, it is clear that the formation of clusters with a specific size and PDI is influenced by both factors (citrate amount and solvent ratio). However, the impact of the DEG/EG ratio is dominating on the size outcome according to the statistical chain analysis.

The obtained F-values for the data confirm the statistical significance of the models. The quadratic model for DLS, SEM, and standard deviation of the size describes well the data. The F-values for the fourth response, the PDI, support the model’s significance. For the last response, there is only a 2.36% chance that an F-value of this magnitude could occur due to noise.

P-values less than 0.0500 indicate that the model terms are significant. In all cases, the observed *p*-values were less than 0.0500, indicating that all of the model’s terms are significant. The lack of fit in the explored data is not significant relative to the pure error and is indicated in the tables above for all of the cases in the respective table (Table 2).

This is a good indication that the models can be fitted to the data. Table 3 summarizes the model statistics for the four responses.

**Figure 5:**
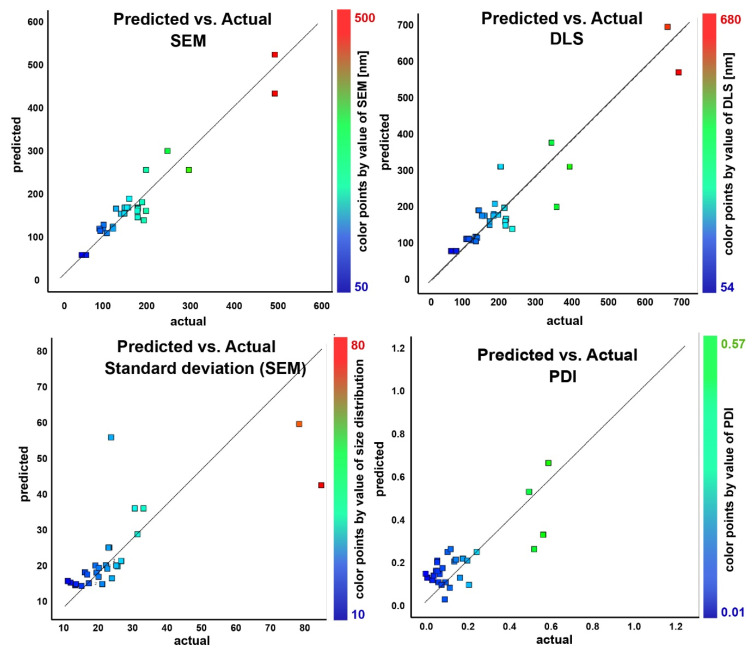
Linear regression plots of the predicted response vs. the obtained response for each experiment. The squares represent the thirty experiments of the model’s training set. The closer the squares are situated to the regression line, the higher the accuracy is of the predicted response.

**Figure 6:**
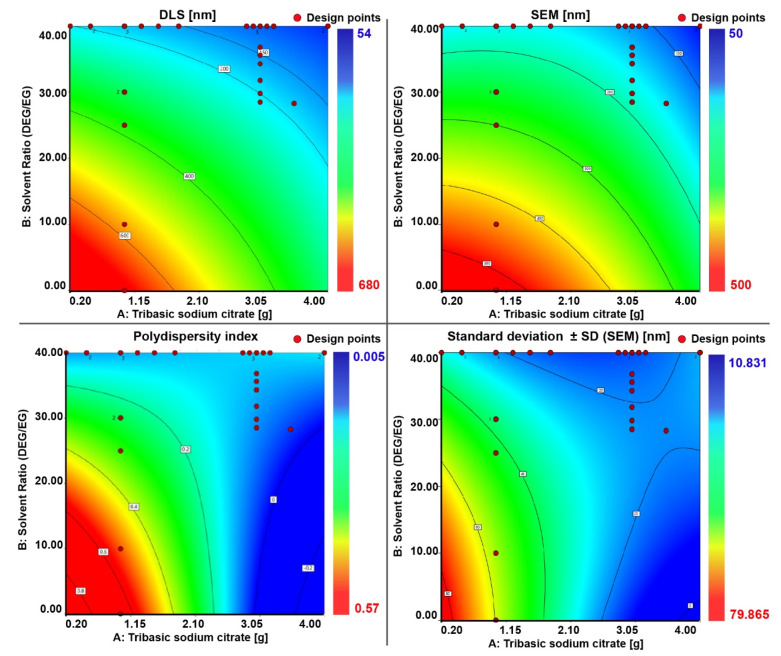
Contour plot for the four responses. The different colors represent different sizes. It is indicated with a specific color which size can be obtained for a specific combination of a certain citrate amount and solvent ratio. The design points represented on the color map represent the experiments we actually performed.

Based on the results obtained from the regression analysis (Figure 5), the optimal experimental levels for independent variables were plotted. The effect of the independent variables (factors) on the dependent variables (responses) for the preparation of the nanocrystal clusters with a targeted particle size and PDI can be read from the contour plots (Figure 6) and from the 3D response surface plots which are illustrated in Figures S11–S14. The contour plot is a 2D illustration of the response for a given amount of citrate and solvent ratio. A change in the response value is represented as a change in color. The design points on the surface represent the experiments that have been performed.

**Figure 7:**
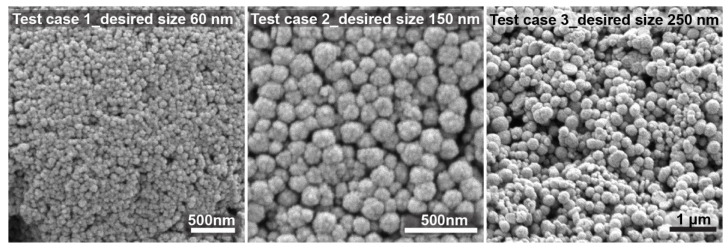
Test runs to prove that the model is working after optimization.

To further validate the model, three test experiments for nanocrystal clusters with specific sizes were performed according to the predicted factors. The size of the nanocrystal clusters can be varied between 50 and 200 nm, with an acceptable PDI between 0.2 and 0.005, depending on the target size. Results above 250 nm were unacceptable because of too high polydispersity. Through numerical optimization procedures, the model was tested by choosing target cluster sizes of 60, 150, and 250 nm. The recipes given by the model to achieve the desired sizes all showed a desirability factor of 1. The desirability function was used as a ranking score for the obtained recipes for the targeted factor. The desirability factor ranges from 0 to 1 (where 1 means a favorable optimization scheme). The algorithm for the desirability function is a multiple response method combining all the responses into a single number for each factor with a given score. The final loop is a scanning of the scored surface for the greatest overall desirability (1 or close to 1). A value of one represents the best score for the given case. Cases with a score of zero indicate that one or more responses collapse outside the scanned surface and are not in the desirable limit factor [41]. The desirability factor being 1 validates the model as being very accurate in its prediction to achieve the desired response. Figure 7 shows SEM images of the nanocrystal clusters obtained from these test experiments. The desired size was achieved in all cases. The first test was chosen to be close to the lower limit of the size range, i.e., 60 nm. The second test case was chosen to be exactly in the middle of the size range, for which the model works very precisely with low PDI. The third case was chosen to be slightly above the size limit of 200 nm, with a desired nanocrystal cluster size equal to 250 nm. It can be observed that test case 3 already shows higher polydispersity, but spherical nanocrystal clusters are still obtained. The experimental results based on the prediction models did not show significant differences from the predicted values, thus confirming the reliability of the developed approach.

To summarize, a combined approach was applied to test the obtained pattern in the data from the experimental design with the aid of multivariate statistics. The proposed cascade scheme reveals some specific relationships between the experimental factors and responses used in the DOE section. Hierarchical cluster analysis (HCA) and non-hierarchical (K-means) clustering methods were used to identify patterns (categories) of similarity between the experimental conditions or responses and the factors involved. The two clustering methods are another way to reveal the partitioning into subgroups of all of the cases (experimental data) based on the responses. The goal of the multivariate statistical analysis and DOE are the following for concrete systems:

1. Finding patterns of similarity between the experiments (objects);
2. Finding relationships between experimental conditions and responses;
3. Defining the classes of magnetic clusters based on a K-means partition distance.

**Figure 8:**
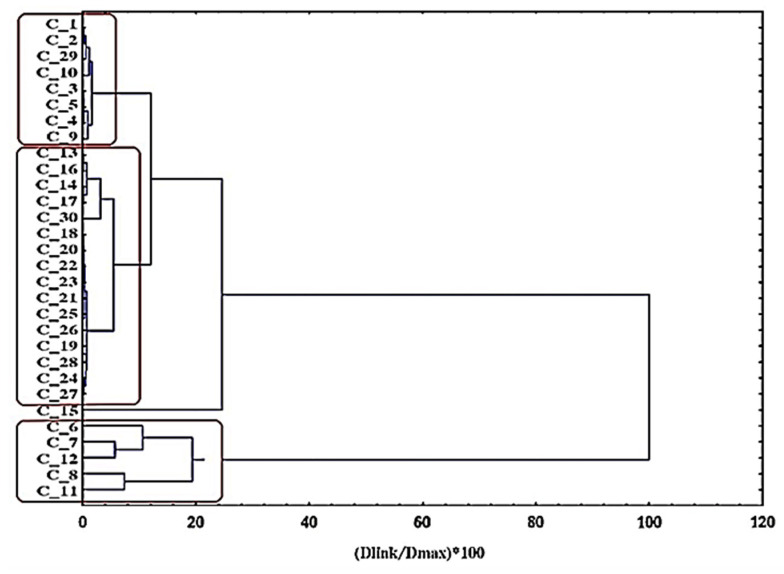
Hierarchical dendrogram for 30 objects (iron oxide nanocrystal clusters) obtained using Ward’s hierarchical clustering method.

The graphical output from the hierarchical cluster analyses (dendrogram) based on a clustering of objects can be seen below (Figure 8). The similarity patterns seen within the nanocrystal clusters are grouped into three categories according to the conditions listed below:

- C1 (MM6–MM8, MM11, and MM12)—five conditions (low citrate levels, low DEG/EG);
- C2 (MM13–MM28 and MM30)—17 conditions (relatively high citrate levels, high DEG/EG);
- C3 (MM1–MM5, MM9, MM10, and MM29)—eight conditions (intermediate citrate levels, high DEG/EG).

Indeed, the dendrogram links the experimental conditions in a logical way, following the same pattern according to the size population partitioning within the data (Figure 8). This clustering corresponds to the hierarchical clustering analyses. In C1, the outliers of the performed experiments are found, meaning the samples that have unacceptably high PDI or sizes higher than 200 nm. C2 predominantly contains the experiments that were run to optimize the model, while C3 mostly contains the experiments that were run to generate the models.

**Figure 9:**
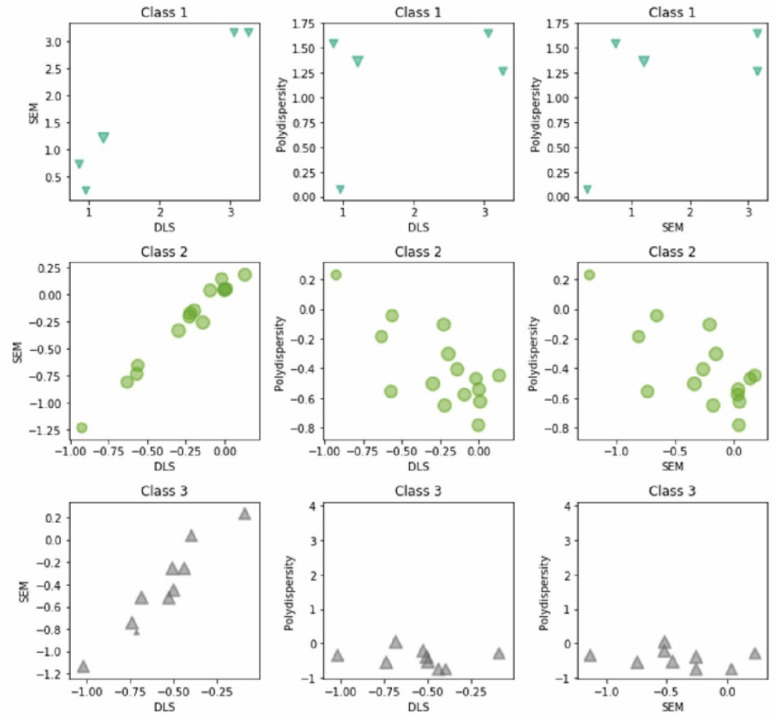
**A** 2D representation of each class obtained by statistical analysis. The axes show the position of each experiment in the space, read out of the K-means plot.

The K-means plot (Figure S15) illustrates how the dependent variables are linked to the respective experimental conditions based on the K-means clustering algorithm. The same pattern is observed as in the case of HCA. The hypothesis of the linkage of size variables implies the formation of three clusters. The next plots are based on the partitioning distances obtained from the K-means. The approach can be easily used for the classification of the data, which are divided with help of the obtained clusters based on their distance inside their classes. The present plot (Figure 9) represents a simple classification procedure that makes it possible to illustrate, on one figure, the grouping of the experimental conditions (three classes) and the sizes of particles presented by the experimental responses, DLS, SEM, and polydispersity, from each of the classes. The procedure is based on the clusters’ distances from the K-means non-hierarchical clustering. The distances between each of the objects within each cluster were determined and are shown in Table S5. The a priori required number of clusters was three due to the preliminary information from hierarchical clustering.

Class 1 contains the cases with larger magnetic nanocrystal clusters. Those objects are defined as outliers of the performed experiments and are represented in the first line of plots. Each experiment is graphically represented with a blue triangle. The polydispersity is very high in each case. On the second line, we can observe the results for Class 2. Class 2 contains the experiments performed to establish the models. The first plot shows a good linear correlation between SEM and DLS sizes. The PDI values measured with DLS are very spread out and varied a lot, and the same trend was observed for the SEM standard deviation of the size. The third class, illustrated in the last set of plots depicted in Figure 9, contains the experiments which were used to optimize the models (increase the accuracy). Again, the sizes from either DLS or SEM are coherent and show a linear correlation. However, an improvement can be seen in the PDI values (which are much lower) due to an increase in accuracy of the predicted factors. Finally, in Class 3, the experiments are those in which the amount of citrate and the solvent ratio were changed simultaneously according to the prediction of the model, providing the optimal recipe for the desired cluster size, showing an almost constant and low PDI and size variance values from SEM. The same information can be represented in a 3D plot, which is found in Figure S16.

## 4. Conclusions

Solvothermal synthesis of magnetic nanocrystal clusters is a flexible and well-established method to prepare water-dispersible monodisperse magnetite nanocrystal clusters with promising applications in biotechnology. To avoid the painstaking trial-and-error procedure normally used to prepare nanocrystal clusters with a desired size and a narrow size distribution, in this work, the synthesis process was analyzed by means of RSM. RSM is a powerful statistical approach which has been used for optimization of the here-used solvothermal synthesis. The objective was to develop a model that can provide the optimal recipe to obtain nanocrystal clusters with a desired size and low PDI. Several experiments were carried out in order to develop the model. An optimized recipe to synthesize nanocrystal clusters with a target size, based on two input factors, namely the citrate concentration and DEG/EG solvent ratio, has been given. The chosen responses of the model are the size of the nanocrystal clusters, measured by either DLS or SEM, the PDI (measured by DLS), and the standard deviation of the SEM size. The latter two quantities are used as indicators of the broadness of the size distribution.

The non-linear structure of the system’s responses followed either a quadratic model or a two-factor interaction model. The statistical significance (ANOVA) as well as non-significant lack of fit showed the efficiency of the models for the set of responses. Based on the developed model and the experimental procedure, the optimum conditions to obtain stable and low-PDI nanocrystal clusters were established and successfully tested on a suitable set of experiments. Process optimization for such a system, where the interactions between the factors are significant for the results, was achieved. 

The developed protocol is an example of a reduction in experimental trial–errors by applying powerful statistical methods such as DOE. Using these approaches hand in hand with the experimental process and sampling distributions can become more time-saving and effective for controlling the probabilistic nature of “blind” experiments with multivariate outcomes. We believe that the same approach can potentially be applied to other nanoparticle synthesis protocols, which are often characterized by a similarly high degree of empiricism. 

## Figures and Tables

**Table 1:** Table showing the complete set of runs with the corresponding responses for the size (dynamic light scattering (DLS) and SEM) and the corresponding polydispersity index (PDI) for DLS and size deviation for SEM measurements.

Performed Run	Amount of Tribasic Citrate (g)	Solvent Ratio (DEG/EG)	DLS Size (nm)	SEM Size (nm)	Standard Deviation of Size in SEM (nm)	Polydispersity Index
MM1	1	1	145	180	24.04	0.01
MM2	0.5	1	129	150	22.12	0.01
MM3	3	1	95	100	20.21	0.06
MM4	1	1	139	150	18.36	0.01
MM5	0.5	1	130	130	21.92	0.06
MM6	0.2	1	344	200	29.82	0.20
MM7	1	0.14	680	500	22.63	0.48
MM8	1	0	650	500	73.87	0.57
MM9	4	1	54	60	25.35	0.11
MM10	1	0.6	190	200	31.46	0.12
MM11	1	0.45	330	249.88	79.87	0.55
MM12	1	0.6	380	299.78	29.07	0.51
MM13	2.8	1	120	100.85	14.6	0.06
MM14	2.9	1	126	122.91	12.9	0.14
MM15	3	1	98.8	91.24	13.11	0.15
MM16	3.1	1	111	93.12	11.59	0.14
MM17	3.2	1	121.1	108.94	10.83	0.18
MM18	3	0.58	200.5	190.67	24.33	0.08
MM19	3	0.60	174.4	160.79	21.23	0.12
MM20	3	0.65	170.8	158.31	21.61	0.04
MM21	1.25	1	204.5	181.29	18.67	0.04
MM22	1.5	1	202.8	180.81	22.84	0.005
MM23	1.8	1	159.7	179.83	16.55	0.05
MM24	3	0.96	159.7	141.84	15.51	0.07
MM25	3	0.80	203.3	180.2	16.18	0.06
MM26	3	0.85	222.5	194.67	19.21	0.08
MM27	3.5	0.55	182.7	149.53	19.29	0.09
MM28	1	1	170	155.26	24.04	0.16
MM29	3	1	103	123.23	20.21	0.2
MM30	4	1	68	50	25.35	0.24

**Table 2:** ANOVA (summary for the four responses) for the Quadratic and 2FI models.

Response 1 (DLS) Quadratic Model				Response 2 (SEM) Quadratic Model			Response 3 (Standard Deviation) Quadratic Model			Response 4 (PDI) 2FI		
Source	F-Value	*p*-Value		F-Value	*p*-Value		F-Value	*p*-Value		F-Value	*p*-Value	
**Model**	31.54	<0.0001	**significant**	53.98	<0.0001	**significant**	5.94	0.001	**significant**	3.72	0.023	**significant**
**Factor A** **Sodium citrate tribasic**	5.19	0.0319		7.4619	0.01162		3.34	0.0801		4.32	0.0475	
**Factor B** **Solvent ratio (DEG/EG)**	14.28	0.0009		25.724	<0.0001		0.5005	0.4861		0.0001	0.9900	
**AB**	1.59	0.2199		1.4949	0.2333		1.95	0.1755		4.8721	0.0363	
**A²**	0.0022	0.9626		3.6993	0.0663		1.7	0.2046				
**B²**	0.0235	0.8793		0.0055	0.9413		0.7639	0.3908				
**Lack of Fit**	1.39	0.3410	**not significant**	1.2125	0.4194	**not significant**	0.18	0.2147	**not significant**	0.1926	0.9980	**not significant**

**Table 3:** Model statistics of the four responses.

Response	R²	Adjusted R²	Predicted R²
1 (DLS size)	0.8679	0.8404	0.6606
2 (SEM size)	0.9183	0.9013	0.7618
3 (SEM size standard deviation)	0.7529	0.6818	0.5578
4 (PDI)	0.8679	0.7253	0.5538

## Data Availability

The data files presented in this study available on request from the corresponding author.

## Supplementary Materials

The following data are available online at https://www.mdpi.com/2079-4991/11/2/360/s1, **Figure S1**: SEM images of performed experiments which were not selected to generate the model because their PDI was too high or their shape not acceptable. The solvent ratio or citrate amount were out of the detected limits for the optimal experimental conditions; Figure S2: Assortment of VSM curves measured for the superparamagnetic nanocrystal clusters. The magnetization was normalized to the iron weight. No hysteresis is observed, confirming superparamagnetism. Figures S3–S6: Diagnostics plots for the statistical properties of the model; Figure S7: DFBETAS for intercept vs. run of the first response (DLS); Figure S8: DFBETAS for intercept vs. run of the second response (SEM); Figure S9: DFBETAS for intercept vs. run of the third response (standard deviation of SEM size data); Figure S10: DFBETAS for intercept vs. run of the fourth response (PDI); Figures S11–S14: 3D surface plots; Figure S15: Plot of means for responses based on supervised clustering; Figure S16: 3D and 2D graphic projection representations of the classification within the dataset based on K-means distance between clusters. Class 1 is represented by blue triangles and consists of the outliers of the performed experiments, while Class 2 is represented by green spheres and contains all the experiments which were performed to build up the model. Finally, the third class is represented by grey triangles and represents the experiments that were run for the numerical optimization of the model. Table S1: Table of coefficients of the corresponding coded factors for response 1 (DLS size), together with an assessment metric for the variance of the model; Table S2: Table of coefficients of the corresponding coded factors for response 2 (SEM size), together with an assessment metric for the variance of the model; Table S3: Table of coefficients of the corresponding coded factors for response 3 (standard deviation of size from SEM), together with an assessment metric for the variance of the model; Table S4: Table of coefficients of the corresponding coded factors for response 4 (PDI, from DLS), together with an assessment metric for the variance of the 2FI model; Table S5: The input matrix based on the distances between clusters obtain from the plot of means.
