# Supplementary material for: Solvothermal Synthesis Combined with Design of Experiments—Optimization Approach for Magnetite Nanocrystal Clusters
Source: Nanomaterials (Basel). 2021 Feb 1;11(2):360. doi: 10.3390/nano11020360 (PMC7912753; doi:10.3390/nano11020360)
Supplement: Supplementary file 1 [file nanomaterials-11-00360-s001.pdf]

nanomaterials

*Supplementary Information*

# Solvothermal Synthesis Combined with Design of Experiments—Optimization Approach for Magnetite Nanocrystal Clusters

**Joelle Medinger, Miroslava Nedyalkova * and Marco Lattuada ***

Department of Chemistry, University of Fribourg, Chemin du Musée 9, 1700 Fribourg, Switzerland;
joelle.medinger@unifr.ch
* Correspondence: miroslava.nedyalkova@unifr.ch (M.N.); marco.lattuada@unifr.ch (M.L.)

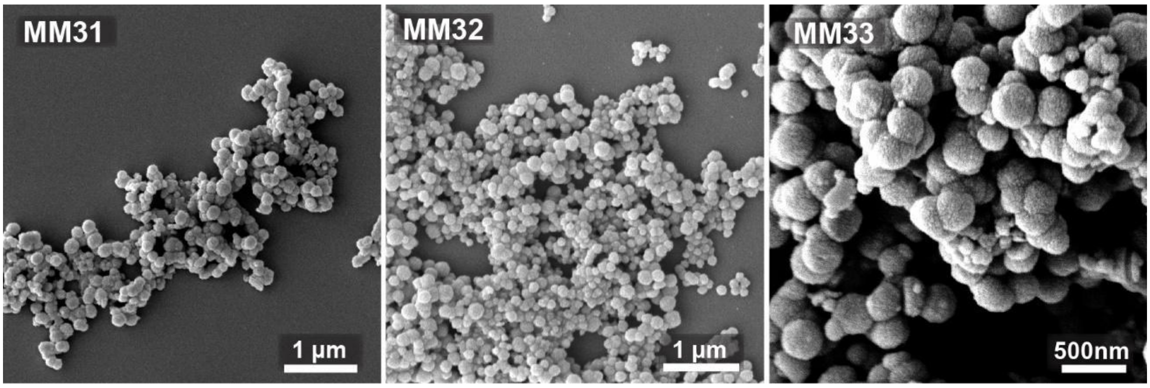


**Figure S1.** SEM images of performed experiments which were not selected to generate the model because their PDI was too high or their shape not acceptable. The solvent ratio or citrate amount were out of the detected limits for the optimal experimental conditions.

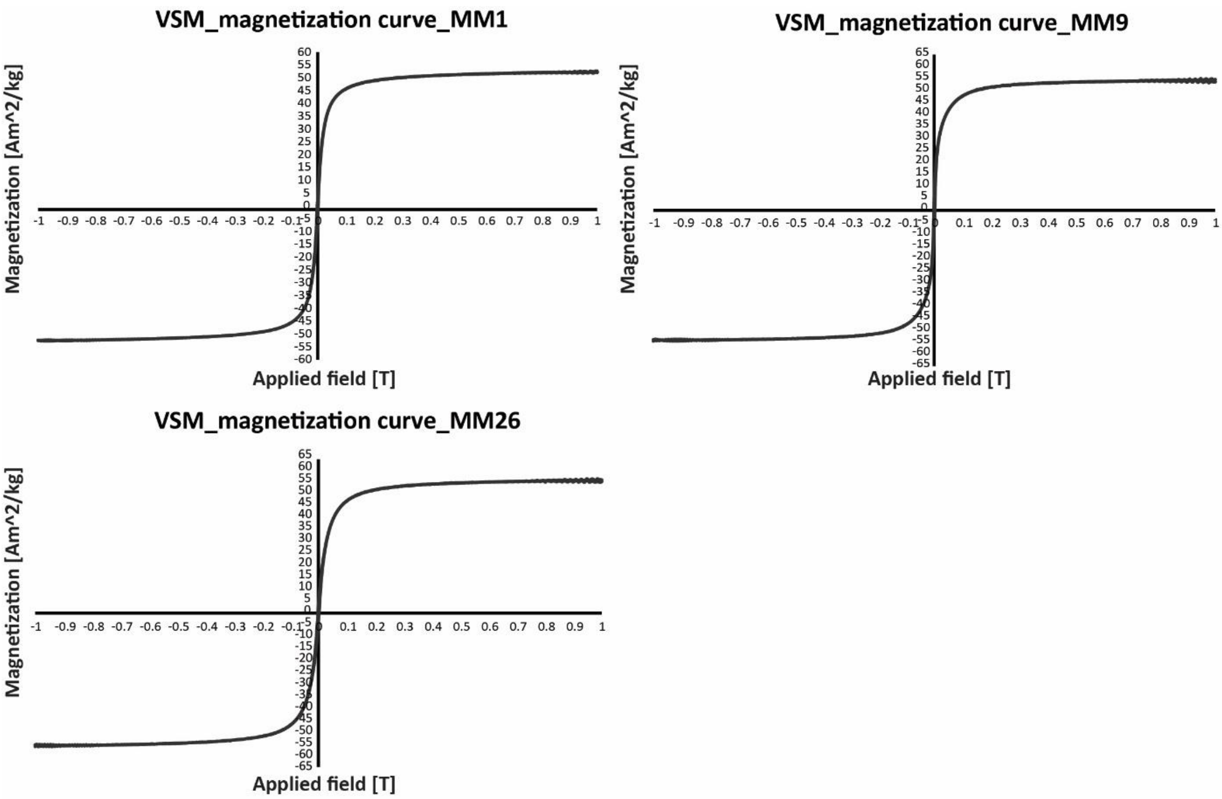


**Figure S2.** Assortment of VSM curves measured for the superparamagnetic nanocrystals clusters. The magnetization was normalized to the iron weight. No hysteresis is observed, confirming superparamagnetism.

Advanced diagnostics scheme for the evaluation of the quadratic model and for the two-factorial interaction model (2FI):

*Quadratic model: Responses (dependent variables): DLS, SEM, Standard deviation (SEM)*

*2FI model: Response (dependent variables): PDI*

Factor Coding in Response Surface Designs

ANOVA: The variance Inflation Factors (VIF) above 10 are acceptable and VIFs above 100 are unacceptable for the model. VIFs above 1000 are not valuable to build up a convenient model. The idea of VIFs diagnostics for the coded factors is to measure how much the variance of the model coefficients increases due to lack of orthogonality in the design. The obtained values of VIF are related to $R_i^2$ (multiple correlation coefficient) by the following: VIF = 1.0/(1-$R_i^2$)

**Table S1. Table of coefficients of the corresponding coded factors for the response 1 (DLS size), together with an assessment metric for the variance of the model**

Response 1: DLS

| Factor | Coefficient Estimate | 95% CI Low | 95% CI High | VIF |
|---|---|---|---|---|
| Intercept | 354.02 | 286.33 | 421.71 | |
| A-Sodium citrate tribasic | −157.98 | −301.07 | −14.90 | 15.31 |
| B-Solvent ratio (DEG/EG) | −203.76 | −315.05 | −92.46 | 5.47 |
| AB | 97.26 | −62.09 | 256.61 | 14.75 |
| $A^2$ | −2.00 | −89.23 | 85.23 | 1.03 |
| $B^2$ | −7.28 | −105.25 | 90.69 | 2.35 |

**Table S2. Table of coefficients of the corresponding coded factors for the response 2 (SEM size), together with an assessment metric for the variance of the model**

Response 2: SEM

| Factor | Coefficient Estimate | 95% CI Low | 95% CI High | VIF |
|---|---|---|---|---|
| Intercept | 299.57 | 262.37 | 336.78 | |
| A-Sodium citrate tribasic | −104.09 | −182.74 | −25.45 | 15.31 |
| B-Solvent ratio (DEG/EG) | −150.34 | −211.51 | −89.16 | 5.47 |
| AB | 51.89 | −35.70 | 139.48 | 14.75 |
| $A^2$ | −44.68 | −92.63 | 3.26 | 1.03 |
| $B^2$ | 1.94 | −51.91 | 55.79 | 2.35 |

**Table S3. Table of coefficients of the corresponding coded factors for the response 3 (standard deviation of size from SEM), together with an assessment metric for the variance of the model**

Response 3: Standard deviation (SEM)

| Factor | Coefficient Estimate | 95% CI Low | 95% CI High | VIF |
|---|---|---|---|---|
| Intercept | 30.71 | 17.75 | 43.67 | |
| A-Sodium citrate tribasic | −24.26 | −51.65 | 3.13 | 15.31 |
| B-Solvent ratio (DEG/EG) | −7.30 | −28.61 | 14.00 | 5.47 |
| AB | 20.63 | −9.87 | 51.14 | 14.75 |
| $A^2$ | 10.55 | −6.15 | 27.25 | 1.03 |
| $B^2$ | −7.94 | −26.70 | 10.81 | 2.35 |

**Table S4. Table of coefficients of the corresponding coded factors for the response 4 (PDI, from DLS), together with an assessment metric for the variance of the 2FI model**

Response 4: PDI

| Factor | Coefficient Estimate | 95% CI Low | 95% CI High | VIF |
|---|---|---|---|---|
| Intercept | 0.1599 | −0.0604 | 0.3803 | |
| A-Sodium citrate tribasic | −0.3892 | −0.7737 | −0.0046. | 9.01 |
| B-Solvent ration (DEG/EG) | 0.0016 | −0.2544 | 0.2575 | 2.36 |
| AB | 0.4662 | 0.0321 | 0.9003 | 8.92 |

**Figure S3 – S6** Diagnostics plots for the Statistical Properties of the Model

Normal Plot of Residuals

The normal probability plot presented below indicates whether the residuals follow a normal distribution, thus follow a straight line. The plots with diagnostics assessment for the model provided below for the four responses DLS, SEM and standard deviation and PDI show a linear distribution, except for four cases (runs – MM6, MM7, MM8 and MM10) which are defined as outlines from the response surface.

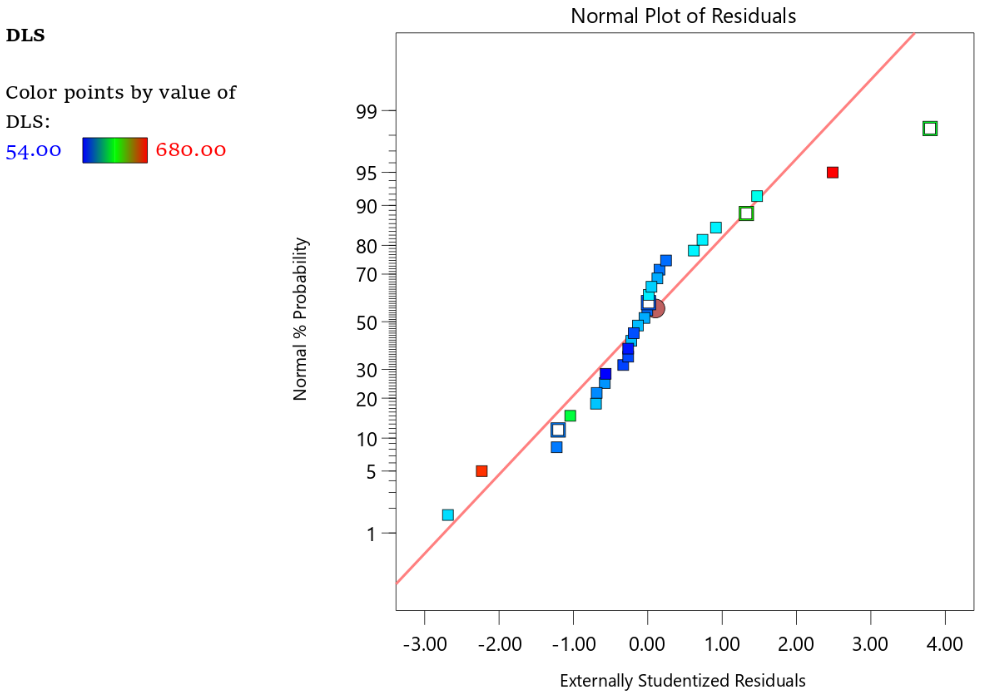


**Figure S3.** Normal probability plot of the residuals for the first response (DLS)

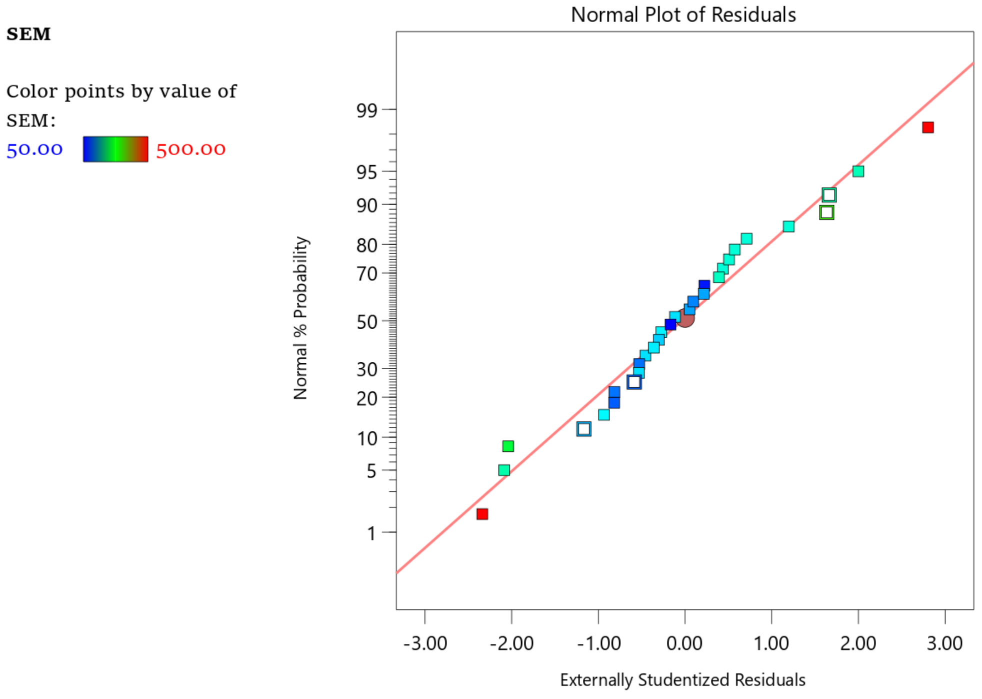


**Figure S4.** Normal probability plot of the residuals for the second response (SEM)

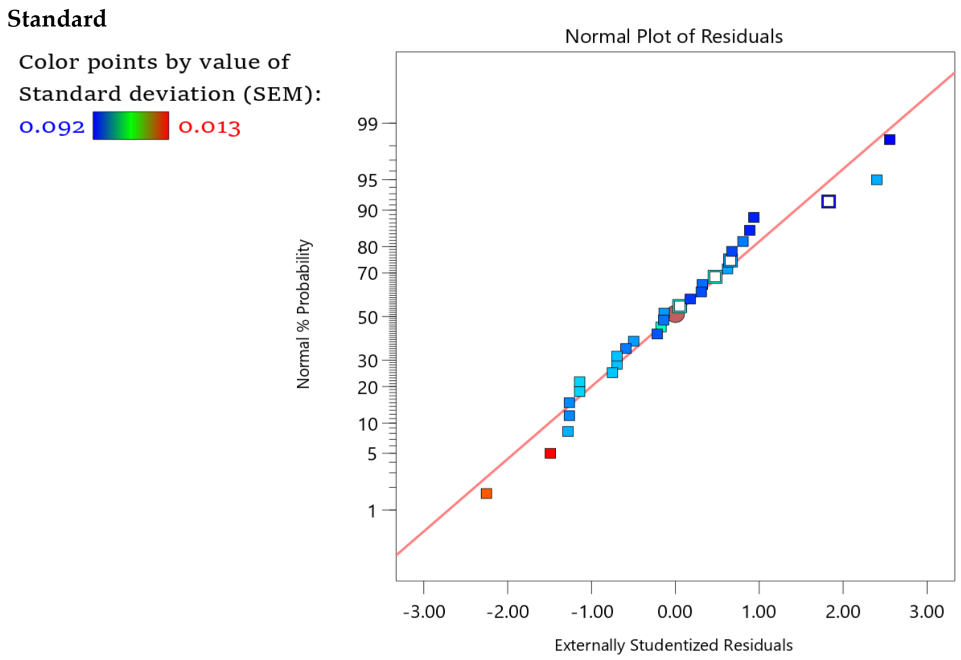


**Figure S5.** Normal probability plot of the residuals for the third response (standard deviation in SEM size data)

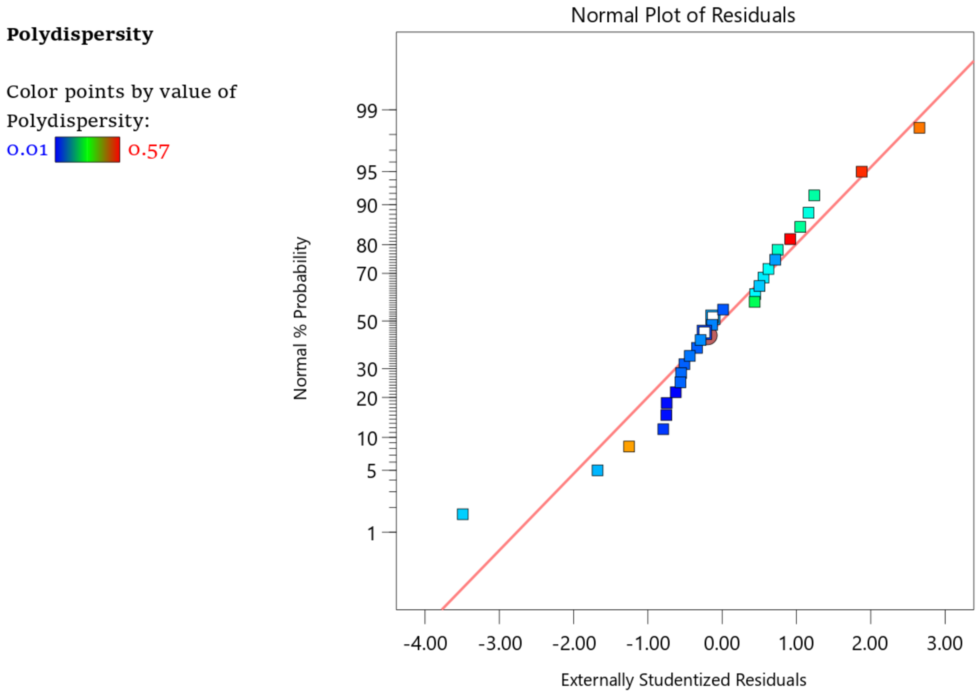


**Figure S6.** Normal probability plot of the residuals for the fourth response (PDI)

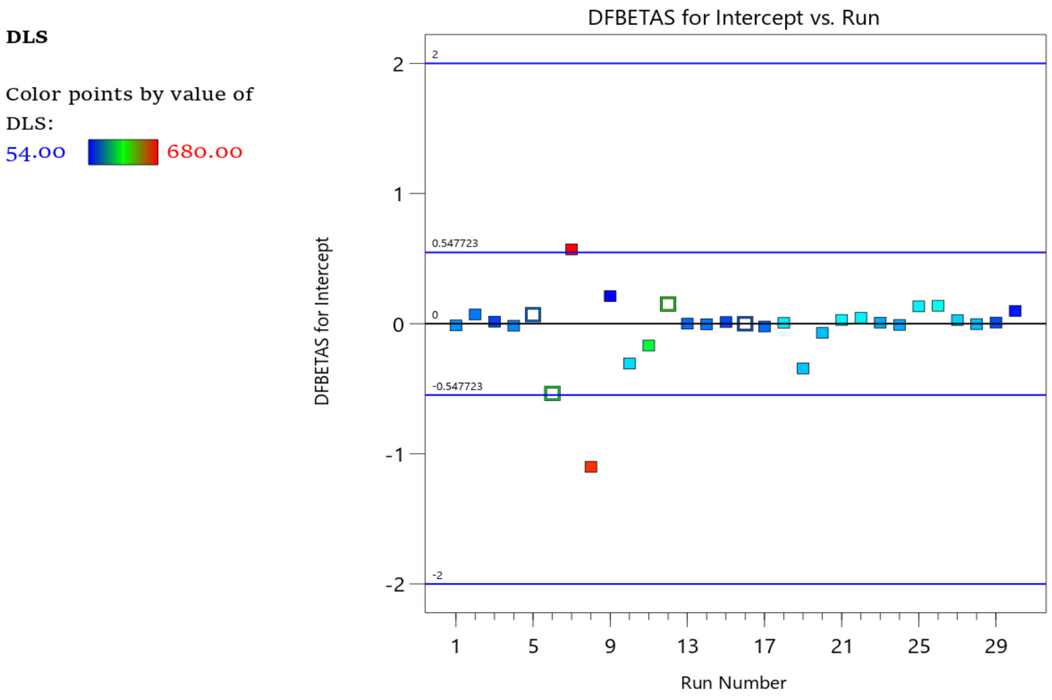


**Figure S7.** DFBETAS for intercept vs. run of the first response (DLS)

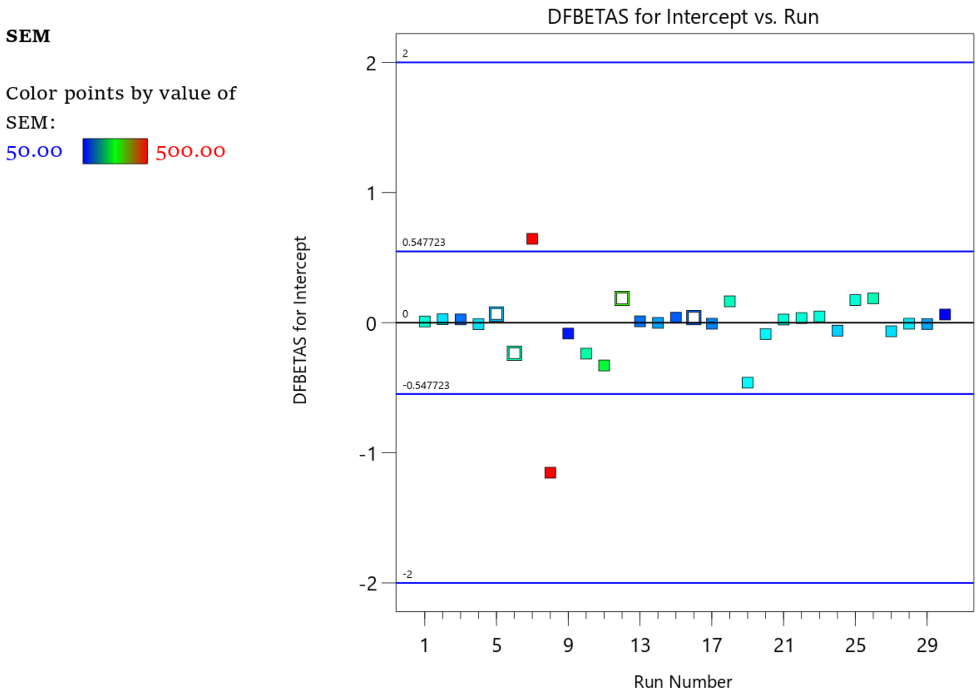


**Figure S8.** DFBETAS for intercept vs. run of the second response (SEM)

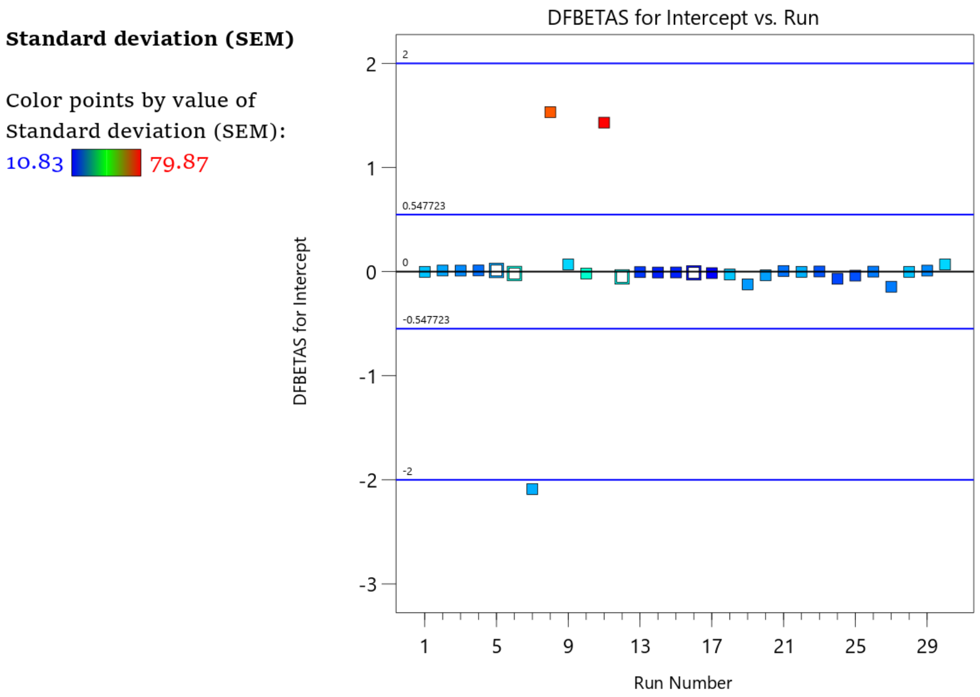


**Figure S9.** DFBETAS for intercept vs. run of the third response (standard deviation of SEM size data)

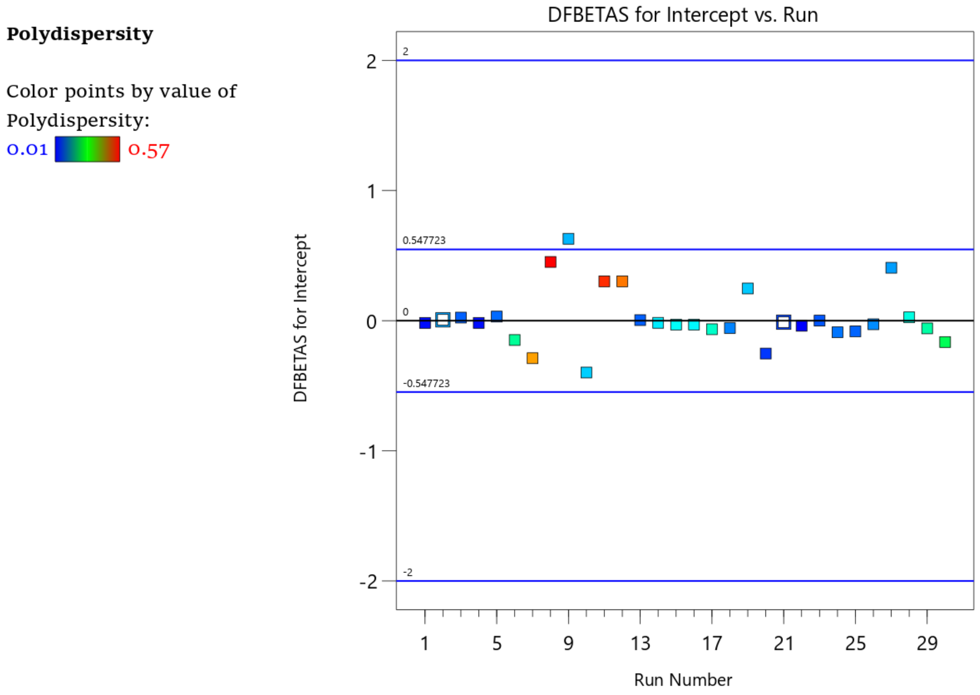


**Figure S10.** DFBETAS for intercept vs. run of the fourth response (PDI)

**Figure S11- S14** 3D surface plots

The three-dimensional representation of the response surface for the four responses were scrutinized below in the following order: DLS, SEM, Standard deviation and PDI. The effect of independent variables on dependent variable of the designed response for the obtained iron oxide nanocrystals clusters' size on 3D surface plots are depicted. The smoothness of the plots according to the change of the factors and responses is well represented in the obtained 3D surface.

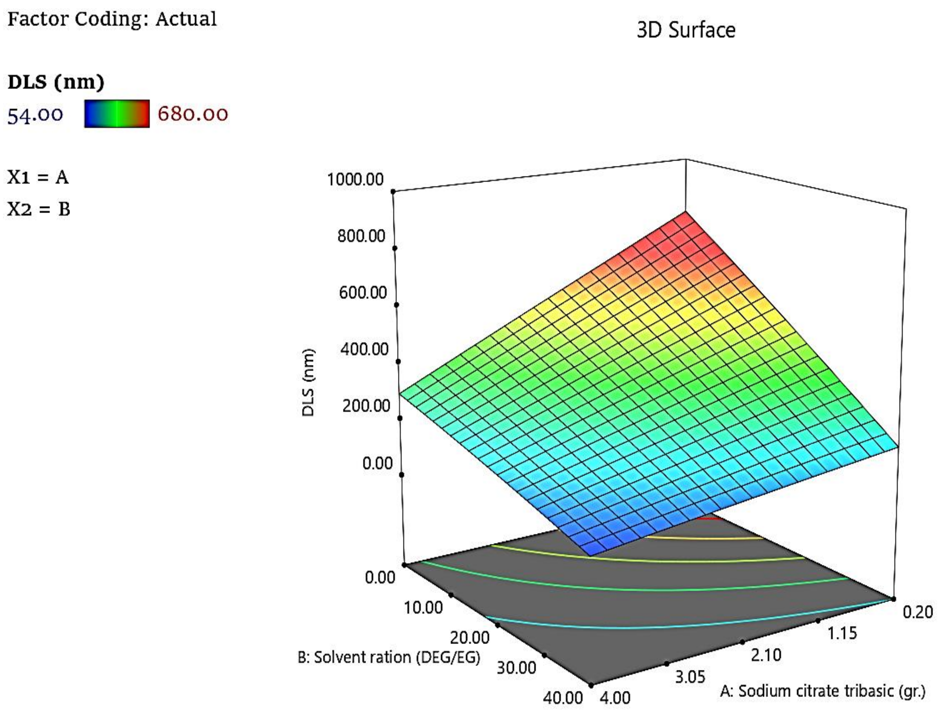


**Figure S11.** 3D surface plot for the first response (DLS). The three axes represented are the quantity of sodium citrate vs the solvent ratio (DEG/EG) with DLS as third axis (response). The colours represent different sizes for the DLS size and are specific for a certain citrate amount and DEG/EG ratio combination.

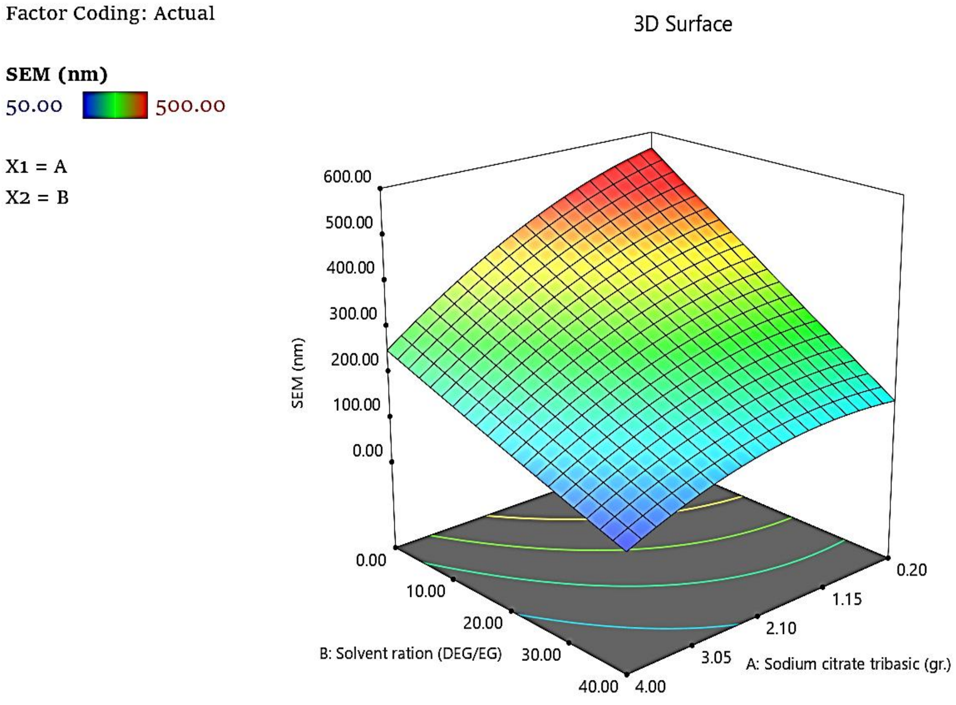


**Figure S12.** 3D surface plot for the second response (SEM). The three axes represented are the quantity of sodium citrate vs the solvent ratio (DEG/EG) with SEM as third axis (response). The colours represent different sizes for the SEM size and are specific for a certain citrate amount and DEG/EG ratio combination.

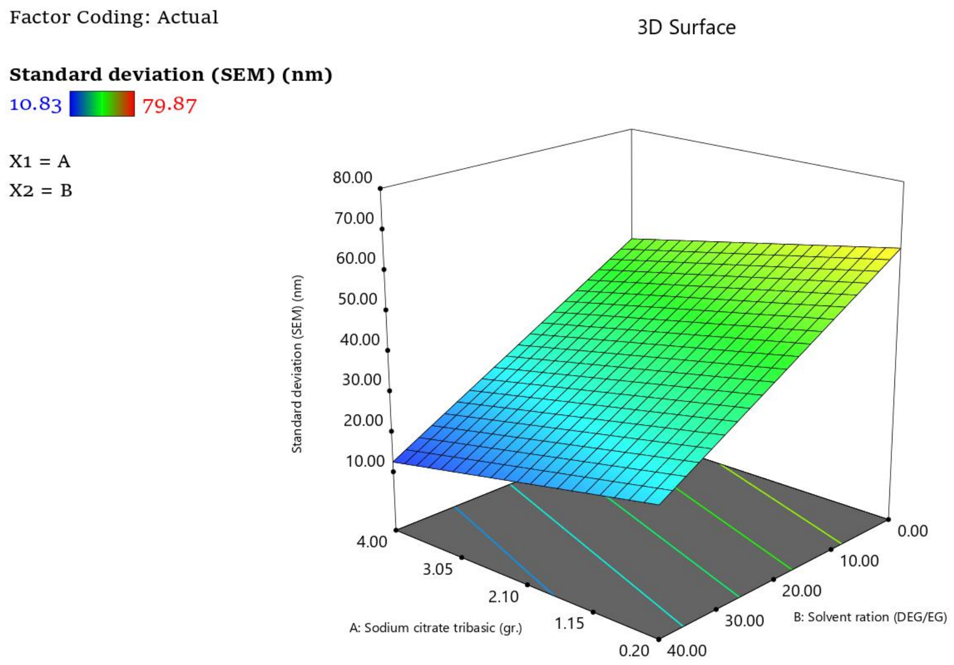


**Figure S13.** 3D surface plot for the third response (standard deviation of SEM size data). The three axes represented are the quantity of sodium citrate vs the solvent ratio (DEG/EG) with SD as third axis (response). The colours represent different standard deviation values and are specific for a certain citrate amount and DEG/EG ratio combination.

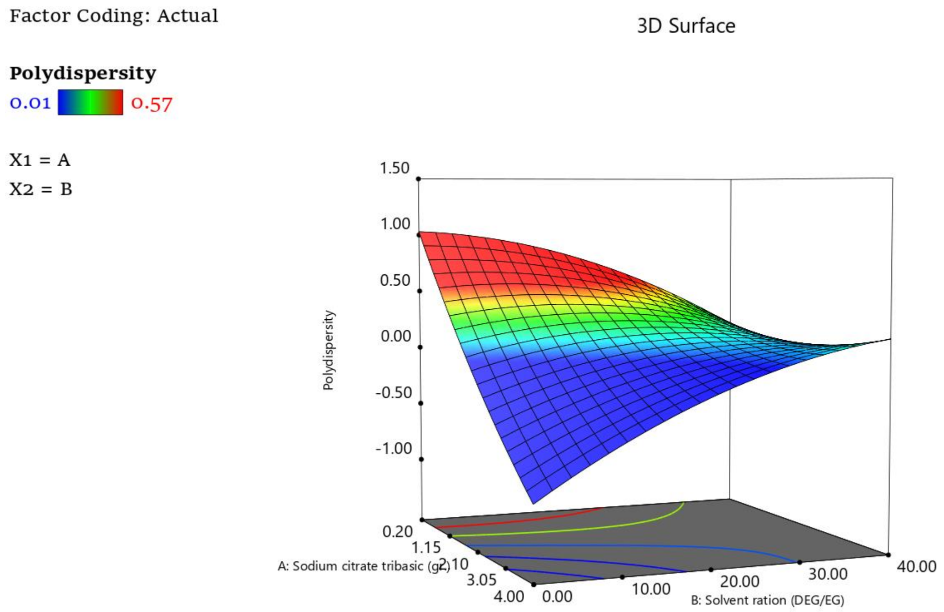


**Figure S14**. 3D surface plot for the fourth response (PDI by DLS). The three axes represented are the quantity of sodium citrate vs the solvent ratio (DEG/EG) with DLS as third axis (response). The colours represent different PDI values and are specific for a certain citrate amount and DEG/EG ratio combination.

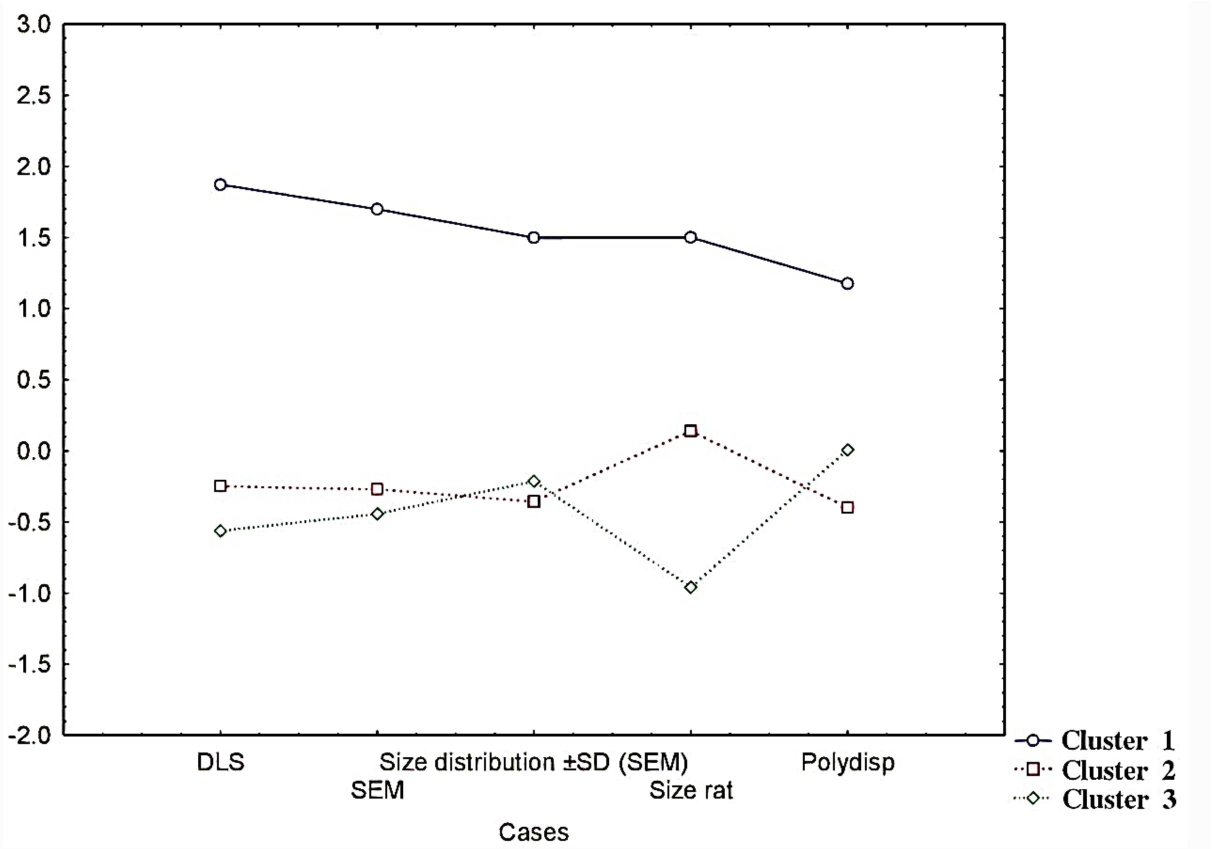


**Figure S15.** Plot of means for responses based on supervised clustering.

**Table S5** - The input matrix based on the distances between clusters obtain from the plot of means (the plot S14)

| ID | Name | DLS | SEM | Polydispersity | Cluster | Cluster distance |
|---|---|---|---|---|---|---|
| 1 | MM1 | −0.397792277 | 0.03805179 | −0.750843664 | 3 | 0.512925 |
| 2 | MM2 | −0.507232463 | −0.2550724 | −0.389917462 | 3 | 0.268133 |
| 3 | MM3 | −0.739792858 | −0.7436128 | −0.551519811 | 3 | 0.298331 |
| 4 | MM4 | −0.438832346 | −0.2550724 | −0.749557703 | 3 | 0.362163 |
| 5 | MM5 | −0.500392451 | −0.4504886 | −0.529658486 | 3 | 0.290357 |
| 6 | MM6 | 0.963370039 | 0.23346794 | 0.070884991 | 1 | 1.304988 |
| 7 | MM7 | 3.26161395 | 3.16471014 | 1.26254157 | 1 | 1.156862 |
| 8 | MM8 | 3.0564136 | 3.16471014 | 1.64404314 | 1 | 1.19348 |
| 9 | MM9 | −1.02023334 | −1.1344451 | −0.349195385 | 3 | 0.43156 |
| 10 | MM10 | −0.089991753 | 0.23346794 | −0.289183903 | 3 | 0.50018 |
| 11 | MM11 | 0.867609876 | 0.72085324 | 1.54116631 | 1 | 1.173887 |
| 12 | MM12 | 1.20961046 | 1.20842304 | 1.36113186 | 1 | 0.715613 |
| 13 | MM13 | −0.568792568 | −0.7352228 | −0.554949039 | 2 | 0.313615 |
| 14 | MM14 | −0.527752498 | −0.5197436 | −0.216312817 | 3 | 0.339051 |
| 15 | MM15 | −0.713800814 | −0.8291947 | 3.86875452 | 3 | 1.787388 |
| 16 | MM16 | −0.630352672 | −0.810811 | −0.186307076 | 2 | 0.396182 |
| 17 | MM17 | −0.561268555 | −0.6563049 | −0.044851439 | 2 | 0.36468 |
| 18 | MM18 | −0.018171631 | 0.14231294 | −0.46921835 | 2 | 0.340732 |
| 19 | MM19 | −0.196695935 | −0.1496183 | −0.302043506 | 2 | 0.165499 |
| 20 | MM20 | −0.221319976 | −0.1738673 | −0.649252797 | 2 | 0.205619 |
| 21 | MM21 | 0.009188416 | 0.05065642 | −0.62353359 | 2 | 0.211948 |
| 22 | MM22 | −0.002439604 | 0.04595021 | −0.782135365 | 2 | 0.278759 |
| 23 | MM23 | −0.092727758 | 0.03638491 | −0.576381711 | 2 | 0.274796 |
| 24 | MM24 | −0.297244106 | −0.3348342 | −0.503510625 | 2 | 0.117453 |
| 25 | MM25 | 0.000980402 | 0.03997835 | −0.542089436 | 2 | 0.206217 |
| 26 | MM26 | 0.132308625 | 0.18138619 | −0.447785678 | 2 | 0.265921 |
| 27 | MM27 | −0.139923838 | −0.2596173 | −0.40577764 | 2 | 0.204273 |
| 28 | MM28 | −0.226791986 | −0.2036966 | −0.104862921 | 2 | 0.230279 |
| 29 | MM29 | −0.685072765 | −0.5165981 | 0.04087925 | 3 | 0.244883 |
| 30 | MM30 | −0.924473173 | −1.2321532 | 0.229486766 | 2 | 0.818973 |

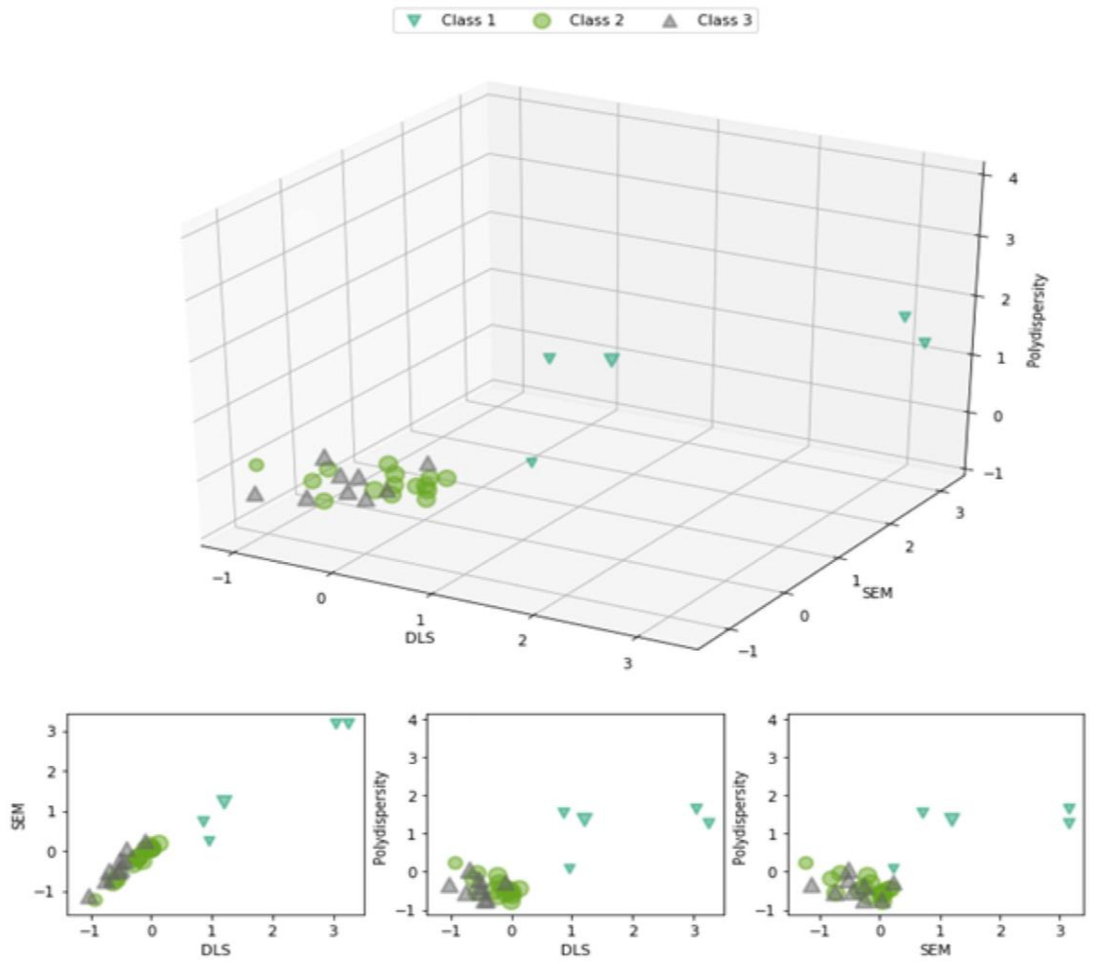


**Figure S16**. 3D and 2D graphic projection representation of the classification within the dataset based on a K-means distance between clusters. Class 1 is represented by blue triangles and consists of the outliers of the performed experiments, while class two, represented by green spheres, contains all the experiments, which were performed to build up the model. Finally, the third class is represented by grey triangles and represents the experiments that were run for the numerical optimization of the model.
